# Risk of infection and adverse outcomes among pregnant working women in selected occupational groups: A study in the Danish National Birth Cohort

**DOI:** 10.1186/1476-069X-9-70

**Published:** 2010-11-15

**Authors:** Maria Morales-Suárez-Varela, Linda Kaerlev, Jin Liang Zhu, Agustín Llopis-González, Natalia Gimeno-Clemente, Ellen A Nohr, Jens P Bonde, Jorn Olsen

**Affiliations:** 1 Unit of Public Health and Environmental Care, Department of Preventive Medicine, University of Valencia, Valencia, Spain; 2 CIBER Epidemiology and Public Health (CIBERESP), Spain; 3 Center for Public Health Research (CSISP), Valencia, Spain; 4 The Danish Epidemiology Science Centre. University of Aarhus, Aarhus, Denmark; 5 Centre for National Clinical Databases South, Dept. of Research and HTA, Odense University Hospital, Denmark; 6 Department of Occupational Medicine, Aarhus University Hospital, Aarhus, Denmark; 7 Department of Epidemiology, School of Public Health, UCLA, Los Angeles, USA

## Abstract

**Background:**

Exposure to infectious pathogens is a frequent occupational hazard for women who work with patients, children, animals or animal products. The purpose of the present study is to investigate if women working in occupations where exposure to infections agents is common have a high risk of infections and adverse pregnancy outcomes.

**Methods:**

We used data from the Danish National Birth Cohort, a population-based cohort study and studied the risk of Infection and adverse outcomes in pregnant women working with patients, with children, with food products or with animals. The regression analysis were adjusted for the following covariates: maternal age, parity, history of miscarriage, socio-occupational status, pre-pregnancy body mass index, smoking habit, alcohol consumption.

**Results:**

Pregnant women who worked with patients or children or food products had an excess risk of sick leave during pregnancy for more than three days. Most of negative reproductive outcomes were not increased in these occupations but the prevalence of congenital anomalies (CAs) was slightly higher in children of women who worked with patients. The prevalence of small for gestational age infants was higher among women who worked with food products. There was no association between occupation infections during pregnancy and the risk of reproductive failures in the exposed groups. However, the prevalence of CAs was slightly higher among children of women who suffered some infection during pregnancy but the numbers were small.

**Conclusion:**

Despite preventive strategies, working in specific jobs during pregnancy may impose a higher risk of infections, and working in some of these occupations may impose a slightly higher risk of CAs in their offspring. Most other reproductive failures were not increased in these occupations.

## Background

An estimated, 320,000 workers worldwide die every year of infection diseases caused by virus, bacteria, and other microorganism [1]. Most pregnant women should be able to continue their work in a workplace free of these reproductive hazards [2], but for women working in close contact with children or sick people, this may be difficult to achieve [3,4]. Some health care workers and childcare workers are often exposed to infectious pathogens at work [5,6]. Furthermore, infections may be a common occupational hazard for food handlers and persons in waste management and sewage work [7].

A number of environmental factors [8], including infections [9], have been implicated in adverse pregnancy outcomes and the teratogenicity of some infections is well documented [3] such as cytomegalovirus (CMV), rubella, parvovirus B19, herpes and toxoplasmosis. Common cold is the most frequently reported maternal infection during pregnancy, but its possible association with adverse pregnancy outcomes is not well studied, and many different viruses are involved [10].

Infections have been reported to cause spontaneous abortion and fetal death [3,11], preterm birth [3,12], intrauterine growth restriction [3,13], and birth defects, including abnormalities of the central nervous system [3,14], ophthalmologic manifestations [3,15], and congenital heart defects [3,16]. The type of adverse effects may vary with gestational age at the time of infection [3,16]. Most infections, however, leave no identifiable trace of damage.

Since infections often are reported with misclassification we base our analyses on occupational titles using high risk occupations as an instrumental variable for exposure. We then check if these occupations are characterized by having a high frequency of infections. Finally we check if those who reported infections in these occupations give birth to children with more reproductive failures.

The aim of this study was to investigate if pregnancy outcomes are associated with occupations involving a potential high risk of infections.

## Methods

### Population

Data were obtained from the Danish National Birth Cohort (DNBC), which is a nationwide study among pregnant women and their offspring. The study is described in detail elsewhere [17]. Between March 1996 and November 2002, pregnant women across Denmark were invited to participate in the study by their general practitioner. Approximately 50% of all general practitioners took part in the recruitment. The exclusion criteria were not having access to a telephone, not speaking Danish well enough to complete the interview, and not intending to carry the pregnancy to term at the first visit. About 60% of those invited chose to participate in the study and signed an informed consent form [18].

A total of 101,047 pregnancies were enrolled in the study, and for 90,301 of these, the women had participated in the first interview of the study that took place at approximately 16 weeks of gestation (interquartile range, 12-20). The data was obtained by computer-assisted telephone interviews. Interviews were missing if the woman could not be reached at the scheduled time or at three additional contact attempts and the interview was cancelled if the woman was no longer pregnant at the time of the interview.

If the women who participated in the first telephone interview had more than one pregnancy during the study period, we included only the first pregnancy and excluded all subsequent pregnancies (n = 2,425). Also, we excluded women if their pregnancy was terminated by an induced abortion (n = 93), hydatidiform mole (n = 42), ectopic pregnancies (n = 24), or if they had multiple birth with no live born infants (n = 3) or if the mother died during pregnancy (n = 1).

### Occupational settings

In the first interview of the study, 83,448 mothers answered questions about their main occupation during pregnancy and three months prior to pregnancy. We excluded unemployed women (n = 18,071).

**Figure 1:**
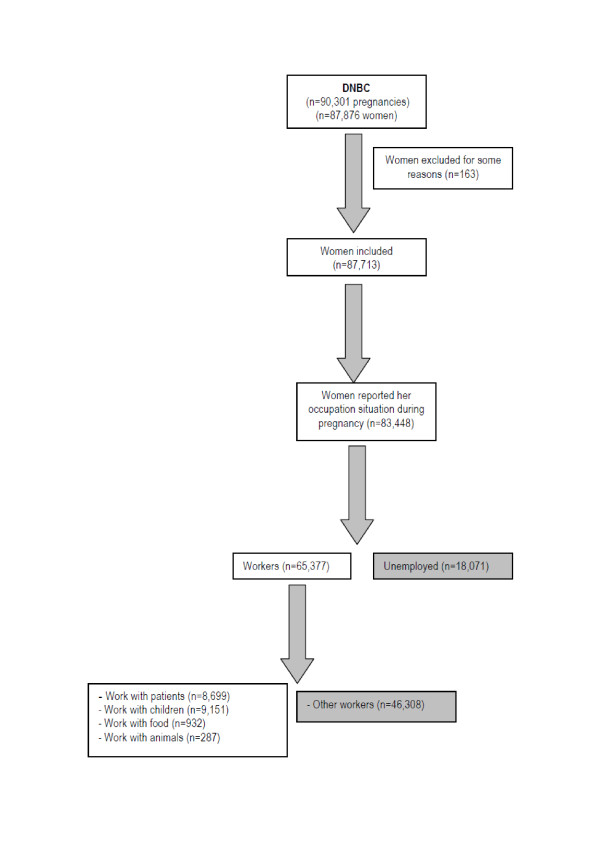
**Selection of the study base**.

The occupation of 65,377 women was later coded according to the Danish Version of International Standard Classification of Occupation (DISCO-88) [19,20]. Women were then categorised to each of the following occupational settings: A) contact with patients (n = 8,699), B) contact with children (n = 9,151), C) contact with food products (n = 932), D) contact with animals (n = 287), and E) the rest were classified as "unexposed workers" (n = 46,308). The groups consisted of A) chemist's assistants (n = 4), physiotherapists (n = 583), dental nurses (n = 444), laboratory technicians (n = 170), doctors (n = 566), auxiliary nurses (n = 2,681) and nurses (n = 4,251), B) child-minders (n = 641), specialist teachers (n = 274), teachers of common school (n = 2,456), upper secondary school teachers (n = 167), care assistant (n = 116), educationalists (n = 3,684) and educationalist assistants (n = 1,813) C) kitchen assistants (n = 842), butchers (n = 90) and D) skilled farm workers (n = 124), slaughter house workers (n = 163), and E) all other workers (n = 46,308) (Figure 1).

### Infections

We collected self-reported information about infections during pregnancy in the second interview carried out approximately at week 30 (interquartile range, 25-32). The women were asked whether she at any point in time during pregnancy 1) had been absent due to illness for more than three days (yes/no) and the number of times she had been absent (never, one-four and more than four times), 2) if she had taken any medications for infectious diseases (yes/no), 3) if she had had an episode of fever (yes/no) and the number of episodes (never, one-two episodes, and more than two episodes), 4) whether she had had diarrhoea (yes/no) and the number of episodes (never, one-four and more than four episodes),5) if she had had cold sore or oral herpes (yes/no), 6) if she had had cold sore or genital herpes (yes/no), 7) if she had had cystitis (yes/no), and 8) if she had had a skin infection (yes/no).

### Pregnancy outcomes

We identified pregnancy outcomes in the National Hospital Discharge Register (NHDR) and the Danish Medical Birth Register by register linkage using the unique personal identification number (civil registration number) which is assigned to all Danish residents at birth. Information about gestational age also came from the National Hospital Discharge Register and was mainly based on ultrasound examination.

We studied miscarriages (late spontaneous abortions and stillbirth), sex ratio (male/female), preterm birth (< 37 weeks of gestation) and very preterm birth (< 34 weeks of gestation), small-for-gestational-age status (< the 10^th ^percentile for the sex - and gestation-specific birth weight in the DNBC by week of gestation) and APGAR (Appearance (skin color), Pulse (heart rate), Grimace (reflex irritability), Activity (muscle tone), and Respiration) [21]) score at five minutes of birth. We also studied congenital anomalies (CAs) according to the codes DQ00-DQ99 from the 10th Revision of International Classification of Diseases (ICD10). We used the European Surveillance of Congenital Abnormalities Classification (EUROCAT) [22] to categorize CAs as minor and major, and excluded chromosome aberrations, genetic syndromes and microdeletions, teratogenic syndrome. We used all CAs and major CAs diagnosed at birth or during the first year of life.

### Other variables

From the first interview, we obtained information about maternal age, parity, pre-pregnancy body mass index (BMI), smoking habits and alcohol consumption during pregnancy, and a history of miscarriage (spontaneous abortions).

Mothers were classified into three categories of socio-occupational status based upon their job title: high, medium or low. Women with a higher level of education (four years beyond high school) or in management positions were classified as "high socio-occupational status". The "medium" category included skilled workers and women with medium-ranged training/level of education, while the "low" category included workers with no formal skilled training [23].

### Statistical Analysis

Odds ratios (ORs) and 95% confidence intervals (95%CI) were used to compare risks of infections in women in occupational settings more prone to infectious exposure (A-D above) with the risks in other women (E). Also, in each working group, we compare risks of adverse pregnancy outcomes in women who suffered some infection with the risks in women who did not suffer any infection during pregnancy.

Multiple logistic regression models were applied to adjust for the following potential confounders: Maternal age: <30,30-34, or >35 years of age; parity: nulliparous women (0) or multiparous women (1+); history of miscarriage: yes or no; pre-pregnancy BMI: underweight (< 18.5), normal weight (18.5-24.9), overweight (25.0-29.9), and obese (30.0+); smoking habit during pregnancy: 0, 1-9, 10-19, or >20 cig./day; alcohol consumption during pregnancy: 0, 0.1 - 19.9, 20.0-39.9 or >40.0 gr./day and socio-occupational status of the mother: high, medium or low.

All the analyses were performed using the SPSS software (version 17.0; SPSS Inc. Chicago III).

The Danish Data Protection Agency approved the study.

## Results

Maternal characteristics are summarized in Table 1. Mothers who worked with patients reported a higher frequency of previous miscarriages and use of fertility treatment prior to the present pregnancy. Compared to the other occupational groups, mothers who worked with food products or with animals were younger and of lower socio-occupational status. They had a higher prepregnancy BMI, were more often smokers, smoked more cigarettes per day, but reported lower alcohol consumption. They reported the lowest history of miscarriages of all groups.

**Table 1:** Characteristics of the study population.

	Work with patients^1^ (N = 8699)		Work with children^2^ (N = 9151)		Work with food^3^ (N = 932)		Work with animals^4^ (N = 287)		Total exposed workers (N = 19069)		Other workers (N = 46308)	
	**N**	**%**	**N**	**%**	**N**	**%**	**N**	**%**	**N**	**%**	**N**	**%**

Maternal age, years												
< 30	4166	48.7	4313	48.0	569	62.3	168	59.2	9216	49.2	19724	43.3
30-34	3089	36.1	3340	37.2	246	26.9	84	29.6	6759	36.1	18425	40.5
35+	1294	15.1	1335	14.9	99	10.8	32	11.3	2760	14.7	7358	16.2
Total	8549	100.0	8988	100.0	914	100.0	284	100.0	18735	100.0	45507	100.0
Parity												
0	3354	38.6	3465	37.9	332	35.7	98	34.1	7249	38.0	17701	38.2
1+	5342	61.4	5684	62.1	599	64.3	189	65.9	11814	62.0	28584	61.8
Total	8696	100.0	9149	100.0	931	100.0	287	100.0	19063	100.0	46285	100.0
Pre-pregnancy body mass index, kg/m^2^												
Underweight (< 18.5)	75	0.9	61	0.7	9	1.1	9	0.1	145	0.8	530	1.3
Normal weight (18.5-24.9)	5183	64.7	4802	58.6	376	44.8	125	48.8	10486	60.6	26081	62.1
Overweight (25.0-29.9)	1949	24.3	2272	27.7	279	33.2	78	30.5	4578	26.5	10997	26.2
Obese/very obese (30.0+)	800	10.0	1064	13.0	176	21.0	53	20.7	2093	12.1	4381	10.4
Total	8007	100.0	8199	100.0	840	100.0	256	100.0	17302	100.0	41989	100.0
Smoking habit												
No	6608	76.0	6779	76.0	619	66.5	182	63.4	14188	74.4	34709	75.0
Yes	2086	24.0	2086	24.0	312	33.5	105	36.6	4872	25.6	11586	25.0
Total	8694	100.0	8694	100.0	931	100.0	287	100.0	19060	100.0	46295	100.0
Smoking habit, cig./day^¥^												
0	6627	76.2	6807	74.4	623	66.9	183	63.8	14240	74.7	34806	75.2
1-9	1193	13.7	1364	14.9	162	17.4	40	13.9	2759	14.5	6483	14.0
10-19	758	8.7	850	9.3	123	13.2	55	19.2	1786	9.4	4241	9.2
20+	116	1.3	127	1.4	23	2.5	9	3.1	275	1.4	766	1.7
Total	8694	100.0	9148	100.0	931	100.0	287	100.0	19060	100.0	46296	100.0
Alcohol consumption, gr./week												
0	6278	72.2	6788	74.2	748	80.3	232	80.8	14046	73.7	32222	69.6
0.1-19.9	149	1.7	157	1.7	5	0.5	3	1.0	314	1.6	744	1.6
20-39.9	1470	16.9	1412	15.4	124	13.3	32	11.1	3038	15.9	8473	18.3
40+	802	9.2	794	8.7	55	5.9	20	7.0	1671	8.8	4869	10.5
Total	8699	100.0	9151	100.0	932	100.0	287	100.0	19069	100.0	46308	100.0
History of miscarriage^†^												
No	7142	82.2	7592	83.0	775	83.2	246	85.7	15755	82.7	38282	82.7
Yes	1550	17.8	1555	17.0	156	16.8	41	14.3	3302	17.3	7984	17.3
Total	8692	100.0	9147	100.0	931	100.0	287	100.0	19057	100.0	46266	100.0
Infertility treatment												
No	8045	92.5	8565	93.6	876	94.0	277	96.5	17763	93.2	42780	92.4
Yes	649	7.5	586	6.4	56	6.0	10	3.5	1301	6.8	3505	7.6
Total	8694	100.0	9151	100.0	932	100.0	287	100.0	19064	100.0	46285	100.0
Work at the time of the 1^st ^interview*												
No	298	3.4	428	4.7	82	8.8	32	11.1	840	4.4	171	0.4
Yes	8400	96.6	8722	95.3	849	91.2	255	88.9	18226	95.6	41039	99.6
Total	8698	100.0	9150	100.0	931	100.0	287	100.0	19066	100.0	41210	100.0
Socio-occupational status												
High	6484	74.5	7386	80.7	213	22.9	49	17.1	14132	74.1	30294	65.5
Medium	2210	25.4	1762	19.3	719	77.1	168	58.5	4859	25.5	14564	31.5
Low	4	0.1	2	0.1	0	0	70	24.4	76	0.4	1421	3.1
Total	8698	100.0	9150	100.0	932	100.0	287	100.0	19067	100.0	46279	100.0

^1^Health care and laboratory workers in hospitals

^2^Day care centres workers and school teacher

^3^Kitchen assistant, waitress and butcher

^4^Skiled farm worker, slaughter house worker

^¥^1 cigarette = 1 cigarette equivalent, 1 cherrot = 2 cigarette equivalent, 1 cigar = 2 cigarette equivalent, 1 pipe = 1.5 cigarette equivalent.

^†^Among singletons

*First tree month of the pregnant

Table 2 shows occupational groups and symptoms of infections. When we adjusted for additional variables (maternal age, parity, history of miscarriage, socio-occupational status, pre-pregnancy BMI, smoking habit and alcohol consumption) we found that pregnant women who work with patients had a higher risk of sick leave for more than three days, medically treated infections, oral herpes and cystitis. Women who worked with children had an increased risk of sick leave for more than three days, an episode of fever, and skin infections during pregnancy. Finally, pregnant women who worked with food products had an excess risk of sick leave for more than three days.

**Table 2:** Crude and adjusted odds ratios (OR) for infections, symptoms and absence from work due to sickness according to occupation.

During the pregnancy	Other workers (N = 46308)		Work with patients^1^ (N = 8699)				Work with children^2^ (N = 9151)				Work with food^3^ (N = 932)				Work with animals^4^ (N = 287)			
	**N**	**%**	**N**	**%**	**ORc** **(95% CI)**	**ORa** **(95% CI)**	**N**	**%**	**ORc** **(95% CI)**	**ORa** **(95% CI)**	**N**	**%**	**ORc** **(95% CI)**	**ORa** **(95% CI)**	**N**	**%**	**ORc** **(95% CI)**	**ORa** **(95% CI)**

Sickness for more leave than 3 days	9160	27.3	2347	38.7	1.68(1.58-1.78)	1.80(1.70-1.90)	2273	34.4	1.39(1.33-1.47)	1.47(1.38-1.56)	218	37.1	1.58(1.34-1.82)	1.43(1.20-1.66)	58	40.3	1.80(1.29-2.31)	1.10(0.80-1.40)
Episodes of sickness for more leave than 3 days																		
1-4	8808	26.3	2239	37.0	1.67(1.57-1.77)	1.79(1.68-1.90)	2165	32.8	1.38(1.30-1.46)	1.45(1.35-1.55)	207	35.3	1.55(1.30-1.85)	1.40(1.17-1.63)	58	40.3	1.86(1.34-2.38)	1.14(0.80-1.48)
5+	301	0.9	96	1.6	2.09(1.66-2.52)	2.19(1.72-2.80)	95	1.4	1.77(1.40-2.24)	1.86(1.45-2.27)	11	1.9	2.41(1.31-3.51)	2.22(1.19-3.25)	0	-	-	-
Any infection with medication	7379	17.3	1510	18.8	1.10(1.04-1.16)	1.09(1.02-1.16)	1563	18.6	1.09(1.03-1.15)	1.05(0.98-1.12)	139	16.4	0.93(0.78-1.08)	0.92(0.76-1.08)	46	18.2	1.06(0.77-1.35)	1.04(0.74-1.44)
Fever	9412	22.2	1904	23.8	1.34(0.97-1.71)	1.05(0.99-1.11)	2086	24.9	1.47(1.06-1.88)	1.10(1.04-1.16)	164	19.3	1.56(1.12-2.00)	0.90(0.74-1.06)	44	17.5	1.12(0.78-1.46)	0.82(0.59-1.05)
Episodes of fever																		
1-2	5124	12.1	1006	12.6	1.04(0.96-1.12)	1.01(0.92-1.10)	1051	12.6	1.04(0.96-1.12)	0.99(0.91-1.07)	87	10.3	0.83(0.66-1.00)	0.85(0.68-1.05)	27	10.8	0.87(0.58-1.16)	0.91(0.60-1.22)
3+	121	0.3	18	0.2	0.80(0.48-1.12)	1.02(0.94-1.10)	18	0.2	0.76(0.46-1.06)	0.99(0.91-1.07)	3	0.4	1.21(0.40-2.02)	0.85(0.68-1.02)	0	-	-	-
Diarrhoea	7140	16.8	1237	15.4	0.90(0.84-0.96)	0.90(0.86-0.94)	1362	16.2	0.96(0.90-1.02)	0.91(0.85-0.97)	124	14.6	0.85(0.70-1.00)	0.85(0.70-1.00)	41	16.2	0.95(0.68-1.22)	0.86(0.60-1.12)
Episodes of diarrhoea																		
1-4	2284	5.4	478	5.9	1.12(1.00-1.24)	1.13(0.99-1.27)	480	5.7	1.07(0.96-1.18)	1.09(0.97-1.21)	47	5.5	1.04(0.77-1.31)	0.88(0.65-1.11)	21	8.3	1.61(1.04-2.18)	1.00(0.61-1.39)
5+	1214	2.8	243	3.0	1.07(0.93-1.21)	1.09(0.94-1.24)	247	2.9	1.03(0.87-1.19)	1.01(0.85-1.17)	31	3.6	1.29(0.90-1.68)	1.26(0.87-1.65)	10	4.0	1.45(0.77-2.13)	1.04(0.52-1.56)
Cold sore or herpes of lip	5184	12.2	1116	13.9	1.16(1.09-1.23)	1.19(1.10-1.28)	1069	12.7	1.05(0.98-1.12)	1.05(0.97-1.13)	116	13.6	1.14(0.93-1.35)	1.15(0.93-1.37)	32	12.6	1.05(0.72-1.38)	0.98(0.66-1.30)
Cold sore or herpes of genital	659	1.5	109	1.4	0.88(0.71-1.05)	0.84(0.63-1.05)	140	1.7	1.08(0.87-1.29)	1.05(0.82-1.28)	13	1.5	0.99(0.57-1.41)	1.33(0.74-1.92)	2	0.8	0.50(0.01-1.00)	0.82(0.20-1.44)
Cystitis	4555	10.7	977	12.2	1.16(1.08-1.24)	1.16(1.10-1.26)	903	10.8	1.01(0.92-1.10)	1.00(0.90-1.10)	85	10.0	0.92(0.73-1.11)	0.78(0.46-1.10)	23	9.1	0.83(0.54-1.12)	0.73(0.31-1.15)
Skin infection	293	0.7	56	0.7	1.01(0.67-1.35)	0.99(0.65-1.33)	85	1.0	1.47(1.15-1.79)	1.50(1.16-1.84)	6	0.7	1.02(0.45-1.59)	1.10(0.48-1.72)	1	0.4	0.60(0.08-1.12)	0.56(0.08-1.04)

^1 ^chemist's assistants, physiotherapists, dental nurses, laboratory technicians, doctors, auxiliary nurses and nurses.

^2 ^child-minders, specialist teachers, teachers of common school, upper secondary school teachers, care assistant, educationalists and educationalist assistants.

^3^Kitchen assistant, waitress and butcher

^4^Skiled farm worker, slaughter house worker

ORc, crude Odds Ratio

ORa^, ^adjusted Odds Ratio for maternal age, parity, history of miscarriage, socio-occupational status, pre-pregnancy body mass index, smoking habit and alcohol consumption.

Table 3 shows no difference between the different occupational groups for the risk of miscarriages, sex ratio, preterm and very preterm birth, and APGAR <7 at five minutes, but the prevalence of CAs was higher among children of women who worked with patients (ORa 1.09, 95% CI: 1.00-1.18 for all live births and ORa 1.10, 95% CI: 1.00-1.20 for all live born singletons), as well as major CAs (ORa 1.11, 95% CI 1.00-1.22). The prevalence of small for gestational age infants was higher among women who worked with food products (ORa 1.33, 95% CI: 1.07-1.59).

**Table 3:** Crude and adjusted odds ratios (OR) for reproductive failures according to occupation.

	Other workers (N = 46308)		Work with patients^1^ (N = 8699)				Work with children^2^ (N = 9151)				Work with food^3^ (N = 932)				Work with animals^4^ (N = 287)				Total exposed (N = 19069)			
	**N**	**%**	**N**	**%**	**ORc** **(IC 95%)**	**ORa** **(IC 95%)**	**N**	**%**	**ORc** **(IC 95%)**	**ORa** **(IC 95%)**	**N**	**%**	**ORc** **(IC 95%)**	**ORa** **(IC 95%)**	**N**	**%**	**ORc** **(IC 95%)**	**ORa** **(IC 95%)**	**N**	**%**	**ORc** **(IC 95%)**	**ORa** **(IC 95%)**

Miscarriage	427	0.9	86	1.0	1.07(0.85-1.29)	1.25(0.79-1.71)	87	1.0	1.03(0.81-1.15)	0.93(0.57-1.25)	11	1.2	1.28(0.70-1.86)	1.15(0.36-1.94)	1	0.3	0.37(0.01-1.00)	-	185	1.0	1.05(0.90-1.20)	1.04(0.74-1.34)
ŧMultiple births	1050	2.3	210	2.5	1.07(0.92-1.22)	1.07(0.92-1.22)	170	1.9	0.82(0.69-0.95)	0.82(0.69-0.95)	16	1.7	0.75(0.46-1.04)	0.76(0.45-1.07)	2	0.7	0.30(0.08-1.20)	0.30(0.07-1.20)	398	2.1	0.92(0.82-1.02)	0.92(0.81-1.03)
^†^Male infant	22740	51.2	4298	51.5	1.01(0.97-1.05)	1.01(0.97-1.05)	4517	51.2	1.00(0.96-1.04)	1.01(0.95-1.07)	441	49.1	0.92(0.81-1.03)	0.92(0.80-1.04)	142	50.4	0.97(0.77-1.17)	1.02(0.80-1.20)	9398	51.3	1.01(0.97-1.05)	1.01(0.97-1.05)
^†^Preterm birth	2297	5.2	420	5.0	0.97(0.87-1.07)	1.00(0.90-1.10)	446	5.1	0.98(0.88-1.08)	0.99(0.89-1.09)	55	6.1	1.20(0.91-1.49)	1.11(0.83-1.39)	15	5.3	1.02(0.60-1.40)	0.98(0.58-1.38)	936	5.1	0.99(0.91-1.07)	1.01(0.92-1.09)
^†^Very preterm birth	292	0.7	37	0.4	0.70(0.48-0.92)	0.54(0.01-1.07)	60	0.7	1.03(0.78-1.18)	0.94(0.45-1.33)	8	0.9	1.36(0.67-2.05)	0.90(0.12-1.68)	3	1.1	1.62(0.51-2.73)	1.23(0.39-2.07)	108	0.6	0.90(0.72-1.08)	0.99(0.78-1.10)
^†^Small-for-gestational age	4231	9.6	769	9.8	0.96(0.89-1.03)	1.01(0.93-1.09)	787	9.0	0.93(0.86-1.00)	1.02(0.93-1.11)	110	12.3	1.32(1.09-1.55)	1.33(1.07-1.59)	24	8.5	0.88(0.58-1.18)	0.84(0.55-1.13)	1690	9.3	0.97(0.91-1.03)	1.03(0.97-1.09)
Apgar <7 at 5 min	632	1.4	109	1.3	0.92(0.75-1.09)	0.94(0.76-1.12)	117	1.3	0.93(0.77-1.09)	0.97(0.80-1.14)	10	1.1	0.78(0.42-1.14)	0.70(0.36-1.04)	3	1.1	0.76(0.24-1.28)	0.73(0.23-1.23)	239	1.2	0.92(0.80-1.04)	0.94(0.80-1.08)
ŧAll congenital anomalies	2262	4.9	455	5.2	1.08(0.97-1.19)	1.09(1.00-1.18)	427	4.7	0.95(0.86-1.04)	0.98(0.88-1.08)	47	5.0	1.04(0.77-1.31)	1.08(0.80-1.36)	11	3.8	0.77(0.42-1.12)	0.65(0.30-1.00)	940	4.9	1.01(0.94-1.08)	1.03(0.95-1.11)
ŧ^†^All congenital anomalies	2158	4.8	437	5.2	1.08(0.99-1.17)	1.10(1.00-1.20)	417	4.7	0.97(0.87-1.07)	1.00(0.89-1.11)	45	5.0	1.03(0.76-1.30)	1.08(0.79-1.37)	11	3.8	0.79(0.43-1.15)	0.68(0.35-1.01)	910	5.0	1.03(0.95-1.11)	1.04(0.96-1.12)
^†^¶"Major" congenital anomalies	1839	4.1	374	4.5	1.09(0.97-1.21)	1.11(1.00-1.22)	337	3.8	0.92(0.82-1.02)	0.95(0.84-1.06)	37	4.1	1.00(0.71-1.29)	1.04(0.74-1.34)	11	3.8	0.94(0.51-1.37)	0.79(0.40-1.18)	759	4.1	1.01(0.92-1.10)	1.03(0.94-1.12)
¶Multiple major congenital anomalies	455	1.0	89	1.0	1.04(0.83-1.25)	1.09(0.85-1.33)	81	0.9	0.90(0.71-1.09)	1.00(0.77-1.23)	11	1.2	1.20(0.66-1.74)	1.28(0.70-1.86)	4	1.4	1.42(0.60-2.24)	1.34(0.49-2.19)	185	1.0	0.99(0.83-1.15)	1.06(0.90-1.22)

^1^Chemist's assistants, physiotherapists, dental nurses, laboratory technicians, doctors, auxiliary nurses and nurses.

^2^Child-minders, specialist teachers, teachers of common school, upper secondary school teachers, care assistant, educationalists and educationalist assistants.

^3^Kitchen assistant and butcher

^4^Skiled farm worker, slaughter house worker

^¥^Adjusted for maternal age, parity, history of miscarriage, socio-occupational status, pre-pregnancy body mass index, smoking habit and alcohol consumption (as well as for sex of child for preterm and very preterm birth).

ŧAmong live births.

^†^Among singletons.

¶EUROCAT Classification.

In table 4 we analyse if those who reported infections in these "exposed occupations" had children with more reproductive failures. We observe no association between reportly of some infection during pregnancy and the risk of reproductive failures in the exposed groups. However, in women who work with animals, the prevalence of CAs was slightly higher among children of women who suffered one or more infections (ORa 2.93, 95% CI: 1.24-4.62 for all live births and ORa 2.80, 95% CI: 1.23-4.37 for all live born singletons), as well as major CAs (ORa 2.80, 95% CI: 1.23-4.37).

**Table 4:** Odds Ratios for pregnancy outcomes according to infections during pregnancy in exposed occupational groups.

	Miscarriage*		Preterm birth		Very preterm birth		Small for gestational age		Apgar <7 at 5 min		All congenital anomalies (among live births)		All congenital anomalies (among live singletons)		Major congenital anomalies (among live singletons)		Multiple major congenital anomalies	
	**ORc** **(IC95%)**	**ORa**^¥^ **(IC95%)**	**ORc** **(IC95%)**	**ORa**^¥^ **(IC95%)**	**ORc** **(IC95%)**	**ORa**^¥^ **(IC95%)**	**ORc** **(IC95%)**	**ORa**^¥^ **(IC95%)**	**ORc** **(IC95%)**	**ORa**^¥^ **(IC95%)**	**ORc** **(IC95%)**	**ORa**^¥^ **(IC95%)**	**ORc** **(IC95%)**	**ORa**^¥^ **(IC95%)**	**ORc** **(IC95%)**	**ORa** **(IC95%)**	**ORc** **(IC95%)**	**ORa**^¥^ **(IC95%)**

**Work with patients**																		
Infection																		
No	1(ref.)	1(ref.)	1(ref.)	1(ref.)	1(ref.)	1(ref.)	1(ref.)	1(ref.)	1(ref.)	1(ref.)	1(ref.)	1(ref.)	1(ref.)	1(ref.)	1(ref.)	1(ref.)	1(ref.)	1(ref.)
Yes	1.29(0.60-1.98)	0.35(0.01-1.00)	1.15(0.92-1.38)	1.17(0.93-1.41)	1.17(0.46-1.88)	1.03(0.40-1.66)	1.04(0.89-1.19)	1.06(0.90-1.22)	0.92(0.60-1.24)	0.99(0.64-1.46)	0.98(0.80-1.16)	1.03(0.84-1.23)	0.99(0.80-1.18)	1.04(0.84-1.24)	1.01(0.81-1.21)	1.07(0.85-1.29)	1.16(0.73-1.59)	1.29(0.80-1.78)
**Work with children**																		
Infection																		
No	1(ref.)	1(ref.)	1(ref.)	1(ref.)	1(ref.)	1(ref.)	1(ref.)	1(ref.)	1(ref.)	1(ref.)	1(ref.)	1(ref.)	1(ref.)	1(ref.)	1(ref.)	1(ref.)	1(ref.)	1(ref.)
Yes	1.53(0.74-2.32)	1.49(0.34-2.64)	0.95(0.77-1.13)	0.92(0.73-1.11)	0.86(0.38-1.34)	0.72(0.30-1.14)	1.04(0.89-1.19)	1.00(0.84-1.16)	0.92(0.62-1.22)	0.95(0.63-1.27)	0.94(0.76-1.12)	0.95(0.76-1.14)	0.93(0.75-1.11)	0.94(0.75-1.13)	0.99(0.78-1.20)	1.00(0.78-1.22)	1.09(0.67-1.47)	1.12(0.68-1.56)
**Work with food**																		
Infection																		
No	1(ref.)	1(ref.)	1(ref.)	1(ref.)	1(ref.)	1(ref.)	1(ref.)	1(ref.)	1(ref.)	1(ref.)	1(ref.)	1(ref.)	1(ref.)	1(ref.)	1(ref.)	1(ref.)	1(ref.)	1(ref.)
Yes	1.19(0.20-2.18)	-	0.86(0.46-1.26)	0.84(0.43-1.25)	1.05(0.07-2.03)	0.86(0.03-1.69)	1.35(0.87-1.83)	1.30(0.81-1.79)	0.79(0.18-1.40)	1.17(0.22-2.12)	1.01(0.55-1.47)	1.04(0.55-1.53)	1.06(0.57-1.55)	1.11(0.57-1.65)	1.35(0.68-2.02)	1.43(0.69-2.17)	1.27(0.39-2.15)	1.18(0.34-2.02)
**Work with animals**																		
Infection																		
No	1(ref.)	1(ref.)	1(ref.)	1(ref.)	1(ref.)	1(ref.)	1(ref.)	1(ref.)	1(ref.)	1(ref.)	1(ref.)	1(ref.)	1(ref.)	1(ref.)	1(ref.)	1(ref.)	1(ref.)	1(ref.)
Yes	-	-	0.88(0.23-1.53)	1.50(0.31-2.69)	-	-	0.87(0.33-1.41)	1.27(0.41-2.13)	1.36(0.17-2.55)	5.76(0.96-10.12)	9.31(1.15-17.47)	2.93(1.24-4.62)	9.41(1.16-17.66)	2.80(1.23-4.37)	9.41(1.16-17.66)	2.80(1.23-4.37)	3.34(0.34-6.68)	0.24(0.01-1.00)
**All exposed groups**																		
Infection																		
No	1(ref.)	1(ref.)	1(ref.)	1(ref.)	1(ref.)	1(ref.)	1(ref.)	1(ref.)	1(ref.)	1(ref.)	1(ref.)	1(ref.)	1(ref.)	1(ref.)	1(ref.)	1(ref.)	1(ref.)	1(ref.)
Yes	1.34(0.54-2.24)	0.92(0.32-1.52)	1.03(0.89-1.17)	1.02(0.88-1.14)	0.99(0.54-1.44)	0.86(0.46-1.26)	1.05(0.95-1.15)	1.04(0.93-1.13)	0.94(0.71-1.17)	1.00(0.75-1.25)	0.99(0.86-1.12)	1.01(0.87-1.15)	0.99(0.85-1.15)	1.01(0.87-1.15)	1.04(0.89-1.17)	1.08(0.92-1.24)	1.16(0.85-1.47)	1.23(0.89-1.57)

^¥^Adjusted for maternal age, parity, history of miscarriage, socio-occupational status, pre-pregnancy body mass index, smoking habit, alcohol consumption (as well as for sex of child for preterm and very preterm birth and occupation in the group of "all exposed".

¶EUROCAT Classification.

*In this variable the sample size is small because many of the women who lost the baby along his pregnancy did not answer the second interview.

## Discussion

Absence from work for more than three days during pregnancy because of illness was more frequent in women who worked with patients, children or food products compared with other women. Women who worked with patients also reported more infectious diseases treated with medication, oral herpes and cystitis episodes. Women who worked with patients also had more frequent fever episodes and skin infections. On the other hand, woman who worked with patients had a higher risk of CAs and woman who worked with food products had a higher risk of having children who were small for gestational age. In the women who work with animals the prevalence of CAs was higher among children of women who suffered some infection than among children of women who did not suffer any infection during pregnancy. In all the other working groups there was no association between the suffering of some infection during pregnancy and the risk of any other adverse pregnancy outcomes.

The higher risk of CAs we found in woman who worked with patients was previously been reported using data from the DNBC [24]. On the other hand, it is known that many people employed in the health care sector are exposed to a wide variety of contagious diseases and the diversity of potentially infectious agents continues to increase [25]. Health professionals however had a higher risk of cytomegalovirus as well as other herpes virus. Some studies show that maternal infected leukocytes cross the placental tissue and amniotic cells, which are swallowed by the foetus and may cause congenital infection [26] and harm the foetal central nervous system, eyes and liver [27]. Cystitis has been reported to be the most common type of infections in pregnant woman [28] not least in the group working with patients. This condition may be responsible for some of the adverse pregnancy outcomes among health care workers [29].

Other studies have shown that day care centre workers and school teachers in close contact with children are more often exposed to infections in the workplace [30]. Infectious agents like Cytomegalovirus and Parvovirus B19 have been shown to frequently affect women who work with children [31], but we did not find an excess risk of any adverse pregnancy outcome in this group.

In the group of women who work with animals, we did not find any indication of a high exposure to infections that affect humans than in the group of other workers. However, we found a higher prevalence of CAs among children of women who reported some infections during pregnancy.

In this large population-based cohort study, we collected information prospectively, had almost complete follow up on more than 83,000 pregnancies, and could carry our extensive confounder adjustment. The impact of the participation rate on the Danish National Birth Cohort was examined in a study among 49,751 women from the source population, including 15,373 participants in the cohort study. This study concludes that its results have been not biased by nonparticipation. These results are reassuring for studies based on the Danish cohort as the present study [18]. However, our study also has weaknesses. Although we adjusted for a number of potential confounders, confounding of the results by other factors related to working with patients or with children is possible (i.e., ionising radiation [32], magnetic fields [33], chemical substances [34], shiftwork [35] or physically demanding work [36]). A high level of sick leave during pregnancy has been reported in jobs with a high risk of infecting others [37]. This may, however, just reflect a lower threshold for sick leaves if women work with other people.

The ad hoc classification of the job titles that we used in this study was not based on a formerly used Job exposure matrix, we used self-reported information about infections, and confounding by other diseases associated may also have had effect on development of CA. We acknowledge that some of the adjusted estimates for rare outcomes were based on small numbers, but the imprecision was reflected in the width of the corresponding 95% confidence intervals. Furthermore, some of the results may be due to chance based on multiple comparisons. Finally, we investigate the miscarriage only after the first interview, which was around 16 weeks of gestation. Exposure leading to early miscarriage or abortion thus cannot be identified in this study and the actual rate of adverse pregnancy outcomes such as CAs may be underestimated if the women had an induced abortion following a positive prenatal test of CA´s.

The purpose of the present study was to investigate I) whether fever or infections during pregnancy were more common in certain job groups, 2) whether the pregnant women having these jobs that entailed a high risk of infection were at increased risk pregnancy outcomes, and 3) whether in these occupational groups were association between the suffering of some infection during pregnancy and the risk of pregnancy outcomes. We did not find any strong indication of excess risk in these occupations. Nor did we find that woman in these occupations who reported infections were at higher risk of having children with complications.

## Conclusions

Despite preventive strategies, working in specific jobs during pregnancy may impose a higher risk of infections, and working in some of these occupations may impose a slightly higher risk of CAs in their offspring. Most other reproductive failures were not increased in these occupations.

## List of abbreviations

BMI: body mass index; CAs: congenital anomalies; CMV: Cytomegalovirus; DNBC: Danish National Birth Cohort; OR: odds ratios; 95%CI: 95% confidence interval.

## Competing interests

The authors declare that they have no competing interests.

## Authors' contributions

MMMSV organized the study and wrote the manuscript. JO and had the original idea for the work, provided expert advice on study organization and contributed to the writing of the manuscript. JPB contributed to the study design and contributed to the writing of the manuscript. LK, JLZ and EAN contributed by interpreting the results and helped to write the manuscript. MMMSV, ALLG and NGC did the data analysis and contributed to the writing of the paper. All the authors read and approved the final manuscript.
